# Positive end-expiratory pressure titration with electrical impedance tomography and pressure–volume curve in severe acute respiratory distress syndrome

**DOI:** 10.1186/s13613-019-0484-0

**Published:** 2019-01-17

**Authors:** Zhanqi Zhao, Mei-Ying Chang, Mei-Yun Chang, Chien-Hung Gow, Jia-Hao Zhang, Yeong-Long Hsu, Inez Frerichs, Hou-Tai Chang, Knut Möller

**Affiliations:** 1 Department of Biomedical Engineering, Fourth Military Medical University, Xi’an, China; 2 Institute of Technical Medicine, Furtwangen University, Villingen-Schwenningen, Germany; 3 Department of Internal Medicine, Far Eastern Memorial Hospital, No. 21, Sec. 2, Nanya S. Rd., Banciao Dist., New Taipei City, 220 Taiwan, ROC; 4 Department of Anaesthesiology and Intensive Care Medicine, University Medical Centre of Schleswig-Holstein Campus Kiel, Kiel, Germany; 5 Department of Critical Care Medicine, Far Eastern Memorial Hospital, No.21, Sec. 2, Nanya S. Rd., Banciao Dist., New Taipei City, 220 Taiwan, ROC; 6 Department of Industrial Engineering and Management, Yuan Ze University, 135 Yuan-Tung Road, Chung-Li Dist., Taoyuan City, 32003 Taiwan, ROC

**Keywords:** Acute respiratory distress syndrome, Electrical impedance tomography, Titration of positive end-expiratory pressure, Pressure–volume curve, Lung protective ventilation strategy

## Abstract

**Background:**

The study objective was to compare titration of positive end-expiratory pressure (PEEP) with electrical impedance tomography (EIT) and with ventilator-embedded pressure–volume loop in severe acute respiratory distress syndrome (ARDS).

**Methods:**

We have designed a prospective study with historical control group. Twenty-four severe ARDS patients (arterial oxygen partial pressure to fractional inspired oxygen ratio, PaO_2_/FiO_2_ < 100 mmHg) were included in the EIT group and examined prospectively. Data from another 31 severe ARDS patients were evaluated retrospectively (control group). All patients were receiving medical care under identical general support guidelines and protective mechanical ventilation. The PEEP level selected in the EIT group was the intercept point of cumulated collapse and overdistension percentages curves. In the control group, optimal PEEP was selected 2 cmH_2_O above the lower inflection point on the static pressure–volume curve.

**Results:**

Patients in the EIT group were younger (*P *< 0.05), and their mean plateau pressure was 1.5 cmH_2_O higher (*P* < 0.01). No differences in other baseline parameters such as APACHE II score, PaO_2_/FiO_2_, initial PEEP, driving pressure, tidal volume, and respiratory system compliance were found. Two hours after the first PEEP titration, significantly higher PEEP, compliance, and lower driving pressure were found in the EIT group (*P *< 0.01). Hospital survival rates were 66.7% (16 of 24 patients) in the EIT group and 48.4% (15 of 31) in the control group. Identical rates were found regarding the weaning success rate: 66.7% in the EIT group and 48.4% in the control group.

**Conclusion:**

In severe ARDS patients, it was feasible and safe to guide PEEP titration with EIT at the bedside. As compared with pressure–volume curve, the EIT-guided PEEP titration may be associated with improved oxygenation, compliance, driving pressure, and weaning success rate. The findings encourage further randomized control study with a larger sample size and potentially less bias in the baseline data.

*Trial Registration* NCT03112512

**Electronic supplementary material:**

The online version of this article (10.1186/s13613-019-0484-0) contains supplementary material, which is available to authorized users.

## Background

Since its first description 50 years ago, acute respiratory distress syndrome (ARDS) has been extensively studied. Despite the recent improvements in disease management, the mortality rate remains high [1]. According to the therapeutic options in the Berlin definition of ARDS, low tidal volume, higher positive end-expiratory pressure (PEEP), and prone position should be applied in severe ARDS [2]. It is widely acknowledged that tidal volume should be set at ~ 6 ml/kg predicted body weight, which can reduce mortality rate compared to high tidal volume [3]. However, an appropriate “higher” PEEP is still uncertain [4, 5]. Individualized PEEP setting is considered useful in reducing lung damage caused by inappropriately high PEEP [6]. A recent study suggested that the driving pressure was associated with mortality [7]. When low tidal volume is selected, the driving pressure depends on the respiratory system compliance (Crs). Therefore, PEEP titration with Crs is reasonable [8]. Other PEEP titration methods include oxygenation [9] and pressure–volume loop [10]. Caramez et al. have compared ten different parameters for setting PEEP following a recruitment maneuver [11]. Statistically significant differences may have not been revealed due to the small number of studied subjects (*n* = 14) and high variation among them. These strategies for setting PEEP aimed at improving oxygenation, increasing alveolar recruitment while limiting hyperinflation; however, they did not significantly reduce mortality. A recent study claimed that a strategy with lung recruitment and titrated PEEP compared with low PEEP increased mortality [12]. With the concerns regarding the study design, methodology, and data analyses, the results of the study are considered questionable [13].

Electrical impedance tomography (EIT) is a noninvasive and radiation-free technique that allows individual, real-time, bedside imaging of the lungs [14]. EIT uses a set of electrodes that are attached around the thorax, while small imperceptible currents are applied, and the resultant voltages are measured. Subsequently, relative impedance changes are reconstructed in the measurement plane [14]. Recent studies highlighted the potential use of EIT for ARDS in PEEP titration [15–20]. These studies proposed EIT-based methods to optimize PEEP setting by maximizing alveolar recruitment and minimizing overdistension. Up to date, there is no prospective study on ARDS patients evaluating the outcome of EIT-guided PEEP titration compared with traditional methods. The effect sizes of the outcome parameters were unknown.

Our hypothesis was that EIT-guided PEEP titration (with compromise between overdistended and collapsed zones), as compared with our routine method (ventilator-embedded pressure–volume loop), improved respiratory mechanics, oxygenation, and other clinical outcomes. The aim of this pilot study was to examine the differences in various clinical outcomes resulting from these two PEEP titration methods. A prospective study with historical control group was designed.

## Methods

The study was approved by the FEMH Ethics Committee in Taiwan (FEMH-105117-E). The present study involves data from our ongoing registry for EIT guiding PEEP titration (clinical trial registration number NCT03112512, https://clinicaltrials.gov/, registered April 13, 2017). Written informed consent was obtained from all patients or their legal representatives prior to the study. A total of 24 consecutive severe ARDS patients (arterial oxygen partial pressure to fractional inspired oxygen ratio, PaO_2_/FiO_2_ < 100 mmHg) were included for the EIT group and examined prospectively. (Demographics are summarized in Table 1.) For the control group, data from severe ARDS patients treated in our ICU in 2016 were included from our database and analyzed (Ethics approval for data analysis FEMH-106094-E. Thirty-one patients met the inclusion criteria, Table 1.) Patients from both groups were not mechanically ventilated before their ICU admission. They were included into the study from the first day of their ICU stay. Detailed demographics and individual diagnoses of all examined patients are summarized in Additional file 1. General exclusion criteria for both groups were the presence of spontaneous breathing, unstable hemodynamics, confirmed or suspected intracranial hypertension, refractory shock, pneumothorax, total ICU stay less than 3 days. Additional exclusion criteria in the EIT group were age < 18 years, pregnancy and lactation period, and any contraindication to the use of EIT (pacemaker, automatic implantable cardioverter defibrillator, and implantable pumps). The initial ventilator settings involved the use of protective ventilation with a tidal volume of 6 ml/kg predicted body weight, permissive hypercapnia. The PEEP was selected according to the ARDSnet PEEP/FiO_2_ table.

**Table 1:** Comparison of demographics between the EIT and control groups

Demographics	EIT group	Control group	*P* value
Age (years)	50.5 ± 13.3	61.5 ± 19.2	< 0.05
Gender (M/F)	15/9	22/9	0.51
Height (cm)	165.6 ± 7.2	163.2 ± 10.2	0.33
Weight (kg)	68.4 ± 17.4	60.5 ± 12.7	0.16

### PEEP titration in the EIT and control groups

An EIT electrode belt, which carries 16 electrodes with a width of 40 mm, was placed around the thorax in the fifth intercostal space, and one reference electrode was placed at the patients’ abdomen (PulmoVista 500, Draeger Medical, Luebeck, Germany). EIT images were continuously recorded at 20 Hz and stored. Respiratory data from the ventilator was transferred to EIT via MEDIBUS connection. The EIT data were reconstructed with the baseline referring to the lowest impedance value measured before PEEP titration started. The data were filtered using a Butterworth fourth-order low-pass filter with a cutoff frequency of 50/min to eliminate impedance changes synchronous with the heart rate.

In the EIT group, an incremental PEEP trial was performed starting at a pressure of 5–8 cmH_2_O with steps of 2 cmH_2_O till the plateau pressure reached 35 cmH_2_O or unstable blood pressure was observed. Then a decremental PEEP trial with steps of 2 cmH_2_O and duration of 2 min followed. EIT data analysis was achieved with a customized software [21]. Two EIT-based parameters were calculated. Regional compliance was computed in all pixels in the lung regions at each PEEP level. Then, cumulated collapse and overdistension percentages were estimated based on the decrease of regional compliance curve during decremental PEEP titration, either toward lower or higher PEEP levels [22]. The PEEP level selected for the patients in the EIT group was the intercept point of cumulated collapse and overdistension percentages curves, providing the best compromise between collapsed and overdistended lung. This approach corresponds to the recommendations published in the original description of this method [22] and the recent consensus statement on chest EIT [14]. If the intercept point occurred between two PEEP steps, the selected PEEP corresponded to the PEEP step toward the lowest global inhomogeneity index, which indicated the degree of homogeneity of ventilation distribution [23].

In the control group, individual optimal PEEP was selected 2 cmH_2_O above the lower inflection point (LIP) on the quasi-static pressure–volume curve, which was obtained with a ventilator-embedded low-flow maneuver (3 l/min). The pressure increase started and ended at 5 cmH_2_O, and the maximum pressure was limited to 40 cmH_2_O). It was constantly checked that no spontaneous breathing or air leakage in the artificial airway was present during the maneuver. Hemodynamics was closely monitored.

**Fig. 1:**
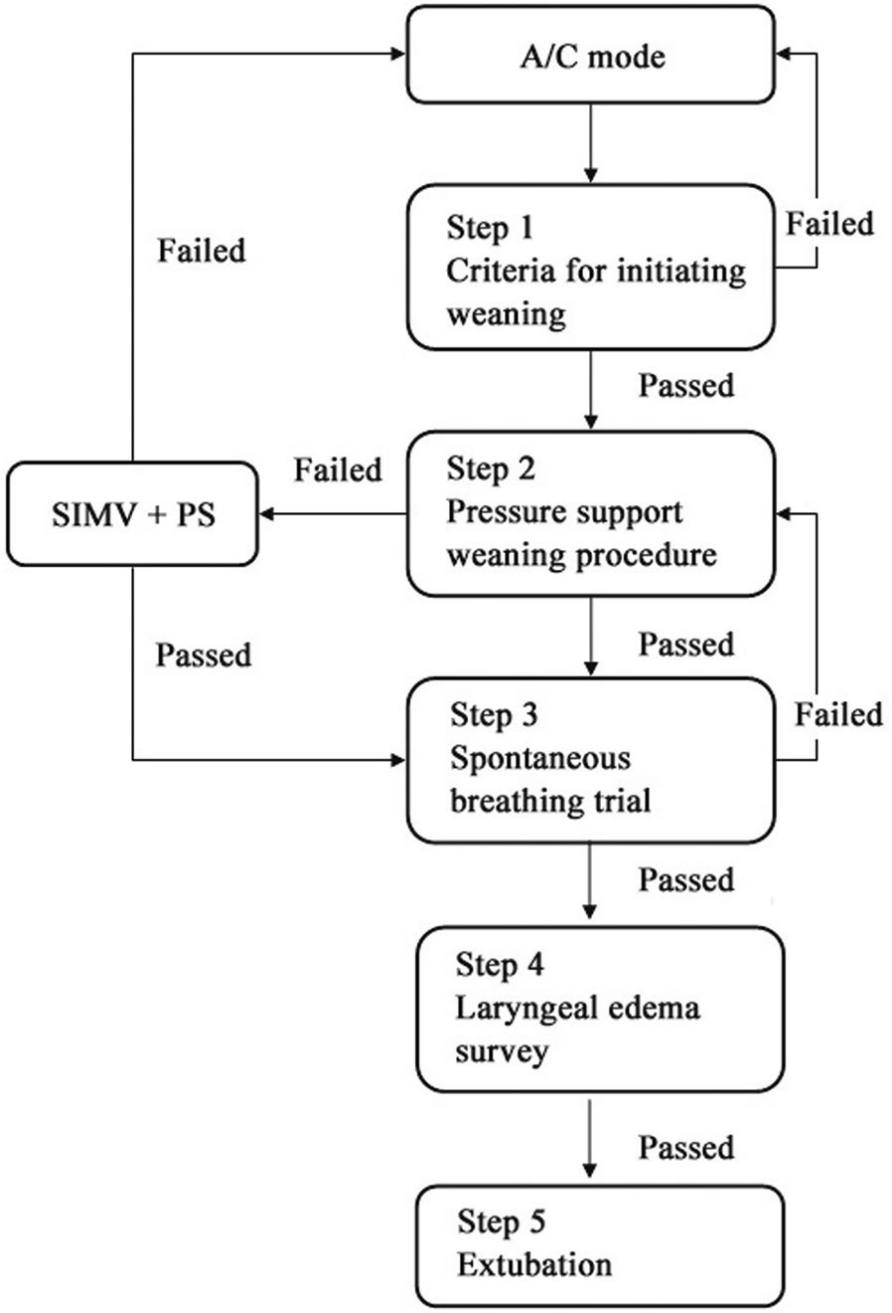
Workflow of internal weaning protocol applied in patients from both EIT and control groups. *A/C mode* assist-control mode. *SIMV* synchronized intermittent mandatory ventilation mode. *PS* pressure support mode

Fentanyl, lorazepam, midazolam, atracurium, cisatracurim, or their combination were used for sedation and neuromuscular blockade. The drug selection, the doses, and the duration of administration were decided by the attending physicians. The ventilation management in both groups involved the use of protective ventilation with a tidal volume of 6 ml/kg predicted body weight, permissive hypercapnia, and preferential use of pressure-limited ventilation modes [24]. After optimal PEEP was selected, and if PaO_2_ remained unchanged, ventilator settings were kept unchanged. If PaO_2_ increased by > 10%, FiO_2_ was slowly adjusted to lower values by 5–10%. Once FiO_2_ reached 0.6, PEEP was decreased in steps of 2 cmH_2_O. Ventilation mode was switched to assist ventilation at the earliest stage. The criteria for initiating weaning, exact weaning procedures, and engagement of spontaneous breathing trial were performed according to our internal weaning protocol (Fig. 1). Pressure support mode was used for weaning. Based on various parameters such as respiratory rate, tidal volume, and blood pressure, the pressure support level was adjusted or weaning procedure was terminated. External continuous positive airway pressure or T-piece methods were used for spontaneous breathing trial. Strategy of spontaneous breathing trial in our center was described in previous studies [25, 26]. After extubation, patients were supported by noninvasive bilevel positive airway pressure mode.

### Data collection and outcome measurements

Demographic characteristics, physiological data, relevant ICU interventions, and radiographic characteristics were collected before the initial PEEP titration after the inclusion of the patients onto the study. Respiratory data before this PEEP titration and 2 h after were collected (except for the Acute Physiology and Chronic Health Evaluation, APACHE II score, which was calculated after 24 h). All patients were followed up to the time of hospital discharge.

The primary outcomes were respiratory mechanics and oxygenation. Exploratory outcome assessments included all-cause hospital mortality (patients discharged to an alternative level of care facility were classified as alive at discharge), presence of barotrauma (pneumothorax, pneumomediastinum, pneumoperitoneum, or subcutaneous emphysema on chest radiograph or chest tube insertions for known or suspected spontaneous pneumothorax), weaning success rate (unassisted breathing without ventilator support for 5 days). As respiratory strategies, nitric oxide, extracorporeal membrane oxygenation (ECMO), and neuromuscular blocking agents (NMBA) were applied after the initial PEEP titration when necessary. The indications and contraindications were based on our internal protocols to ensure that patients from both groups were treated using the same criteria.

### Statistical analysis

Paired *t* test was used to compare respiratory data before and 2 h after PEEP titration. Unpaired *t* test or Chi-square test with Fisher exact test was used to compare the demographics and clinical outcomes between the EIT and control groups where appropriate. Chi-square test was further performed with groups as layer variable to examine whether use of nitric oxide or NMBAs had significant effects on survival rate. When the data was not normally distributed, Wilcoxon signed-rank test or rank-sum test was used instead of *t* test. Log-rank test was performed to assess the differences in numbers of days for hospital survival and weaning success curves of EIT and control group. A *p* value < 0.05 was considered statistically significant. Statistical analysis was performed using SPSS (version 19; IBM Corp. Armonk, NY, USA).

## Results

**Fig. 2:**
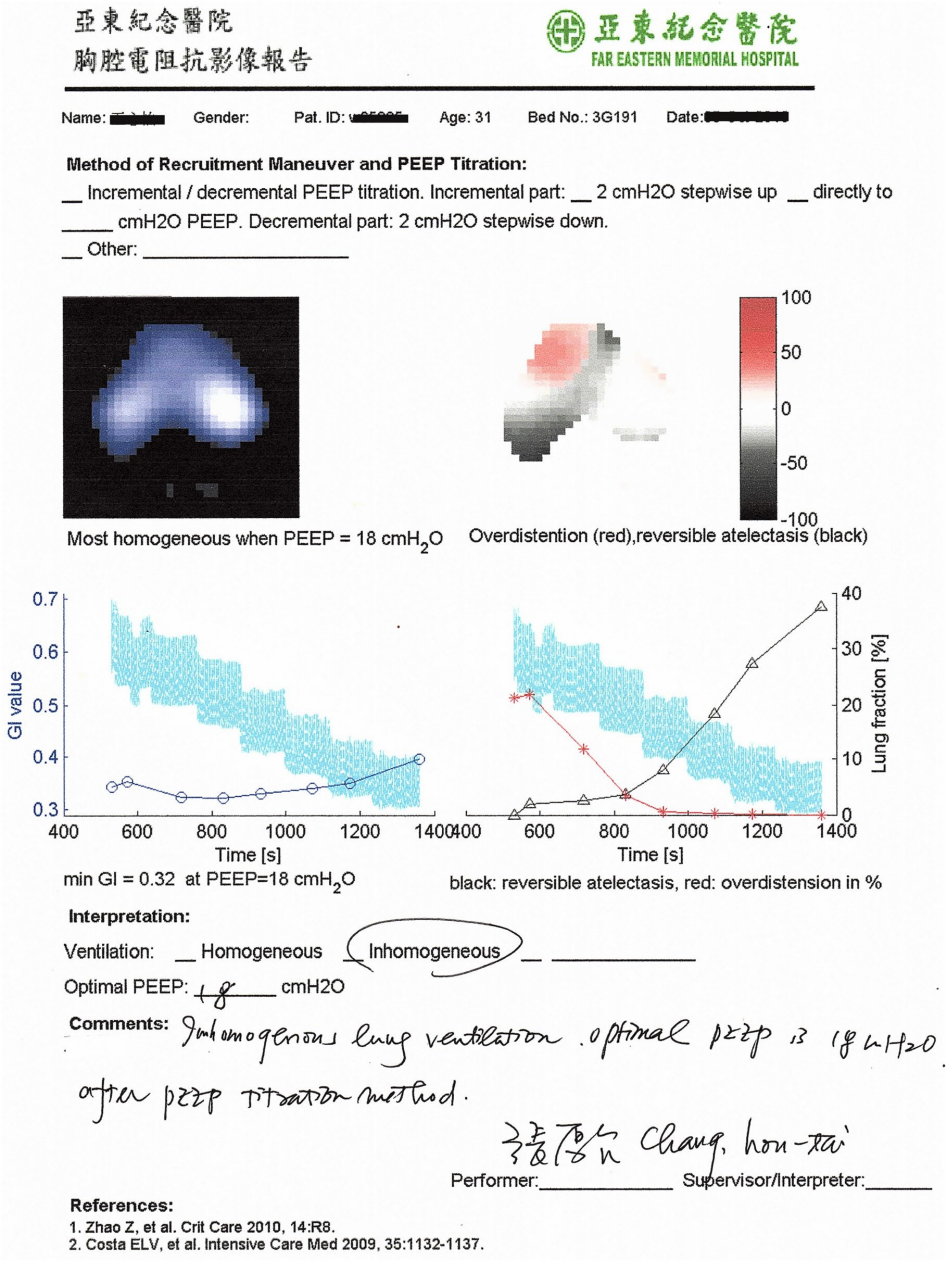
PEEP titration report of an ARDS patient. The PEEP level selected based on EIT was the intercept point of cumulated collapse and overdistension percentages curves (triangle line and asterisk line). If the intercept point occurred between two PEEP steps, the selected PEEP corresponded to the PEEP step toward the lowest global inhomogeneity (GI) index (circle line)

**Fig. 3:**
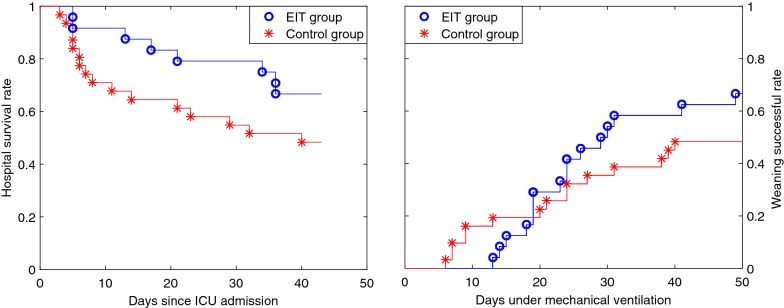
Hospital survival (left) and weaning success curves (right) of EIT group (blue circles) and control group (red asterisk). For the hospital survival curves, day 0 is the day of ICU admission. If a patient survived and was discharged from hospital, he was not censored but counted as survival instead

Patient demographics were comparable between the groups except for age (50.5 ± 13.3 in the EIT group vs. 61.5 ± 19.2 in the control group, *P *< 0.05). Baseline parameters were comparable in the two groups except for plateau pressure (Table 2). Causes of ARDS were diverse in subjects (see Additional file 1). Figure 2 shows a report of the PEEP titration in one of the patients from the EIT group. Table 3 summarizes the outcome parameters compared between the EIT and control groups. Hospital survival and weaning success rates were higher in the EIT group but the differences were not statistically significant. (See also Fig. 3.) Log-rank test also indicated that the differences in the numbers of days were insignificant (*P *= 0.10 and 0.24 for hospital survival (Fig. 3 left) and weaning success curves (Fig. 3 right, respectively). More patients inhaled nitric oxide in the control group (Table 3; *P *< 0.01). Chi-square test indicated that neither inhaled nitric oxide nor NMBAs were associated with survival (*P *= 0.36, 0.48 and 1.00 for inhaled nitric oxide in EIT, control group and overall, respectively; *P *= 1.00, 0.65 and 0.69 for NMBAs in EIT, control group and overall, respectively). No significant differences were found in other ventilation strategies (e.g., ECMO). Driving pressure and Crs were significantly improved in both groups 2 h after PEEP titration (*P *< 0.01; Table 2), but the reduction in driving pressure and the increase in Crs were more pronounced in the EIT group (*P *< 0.01 between groups after PEEP titration). Additionally, APACHE II scores after 24 h were significantly improved in the EIT but not the control group.

**Table 2:** Parameters comparison at baseline and 2 h after the PEEP titration

Parameters	EIT group	Control group	*P* value (between groups)
Baseline			
PaO_2_/FiO_2_ (mmHg)	71.7 ± 16.6	69.7 ± 15.9	0.66
APACHE II	23.2 ± 6.4	23.5 ± 6.9	0.89
PEEP (cmH_2_O)	13.5 ± 1.9	11.5 ± 3.8	0.07
Vt (ml/kg)	6.0 ± 0.8	6.3 ± 1.1	0.27
*P*_driv_ (cmH_2_O)	22.5 ± 2.2	23.0 ± 3.1	0.54
*P*_plat_ (cmH_2_O)	35.9 ± 0.9	34.4 ± 2.4	< 0.01^§^
Crs (ml/cmH_2_O)	16.0 ± 1.8	16.0 ± 2.9	0.91
2 h after (except APACHE II)			
PaO_2_/FiO_2_ (mmHg)	163.7 ± 70.1*	160.0 ± 77.8*	0.86
APACHE II (24 h)	20.6 ± 5.3*	22.7 ± 8.6	0.31
PEEP (cmH_2_O)	17.6 ± 3.6*	13.6 ± 3.6*	< 0.01^§^
Vt (ml/kg)	6.3 ± 0.8	6.5 ± 1.2	0.59
*P*_driv_ (cmH_2_O)	15.1 ± 3.1*	19.1 ± 3.7*	< 0.01^§^
*P*_plat_ (cmH_2_O)	32.7 ± 2.6*	32.6 ± 2.7*	0.98
Crs (ml/cmH_2_O)	25.9 ± 5.9*	20.4 ± 5.3*	< 0.01^§^

PaO_2_/FiO_2_: ratio of arterial partial pressure of oxygen and fraction of inspired oxygen, APACHE: acute physiology and chronic health evaluation, Vt: tidal volume per kilogram predicted body weight, *P*_driv_: driving pressure, *P*_plat_: plateau pressure, Crs: respiratory system compliance

Significant differences compared to baseline values within each group are marked with * (*P *< 0.01). Significant differences between group are marked with ^§^

**Table 3:** Other outcomes and ventilation strategies comparison between two groups

Parameters	EIT group	Control group	*P* value
Outcome			
Hospital survival rate	16/24 (66.7%)	15/31 (48.4%)	0.18
Weaning success rate	16/24 (66.7%)	15/31 (48.4%)	0.18
Barotrauma	0/24 (0%)	2/31 (6.5%)	0.50
Ventilation strategies			
Inhalation of nitric oxide	16/24 (66.7%)	30/31 (96.8%)	< 0.01^§^
ECMO	8/24 (33.3%)	5/31 (16.1%)	0.20
Tracheotomy	5/24 (21%)	4/31 (10%)	0.30
Prone position	1/24 (4%)	0/31 (0%)	0.44
NMBA	23/24 (96%)	26/31 (84%)	0.22

*ECMO* extracorporeal membrane oxygenation, *NMBA* neuromuscular blocking agent

Significant differences are marked with ^§^

## Discussion

In the present study, EIT-guided PEEP titration was prospectively performed in severe ARDS patients. It significantly improved PaO_2_/FiO_2_, APACHE II score, driving pressure and Crs. Further, its clinical outcomes were compared with pressure–volume curve method. EIT-guided PEEP titration is associated with lower driving pressure, higher Crs, higher but not significant hospital survival, and weaning success rates. Results of the preliminary study provided information about the effect sizes of outcome parameters and sample size calculation for future randomized control trials.

To identify individual optimal PEEP remains a popular research topic in the field of intensive care [27, 28]. Besides widely used PEEP titration methods (e.g., Crs, blood gases), new methods such as esophageal pressure [29] and dead space fraction [30] are proposed. Since the application of PEEP aims at maintaining alveoli open, imaging techniques might be the more intuitive methods to select optimal PEEP. As the only bedside tool available, retrospective evaluations of EIT-guided PEEP titration confirmed its feasibility [15, 16, 20, 23, 31]. Two prospective outcome studies were conducted in lavaged pigs, one of them confirming that EIT-guided PEEP selection was superior to the ARDSnet table [17, 32]. The ARDSnet table recommends PEEP according to FiO_2_ levels, which is less individualized than selection based on lung mechanics, blood gases or imaging. In the present study, we chose ventilator-embedded pressure–volume curve as a reference method, as it is routinely used in our department for PEEP titration. A previous study suggested that PEEP setting at 2 cmH_2_O above LIP was more effective in maintaining gas exchange and minimizing injury than PEEP based on adequate oxygenation [33]. In the present study, PaO_2_/FiO_2_, driving pressure, and Crs were improved 2 h after setting the PEEP at LIP + 2 cmH_2_O (Table 2). Previous studies suggested that driving pressure and Crs are risk factors for death [34]. As compared to the control group, the improvements in driving pressure and Crs were more significant in the EIT group. Nevertheless, the effect of a PEEP titration strategy aiming at decreasing driving pressure still has to be demonstrated. Besides, APACHE II scores after 24 h were significantly improved in the EIT but not in the control group. The limitation of using pressure–volume curve to titrate PEEP was that LIP is a global measure, after which regional recruitment would continue to occur. On the other hand, EIT is able to assess regional recruitment better. Although the cross-sectional lens-shaped measuring plane of EIT covers only part of the lung, impedance changes are highly correlated with volume changes of the whole lung [26]. The EIT-based measures used in the present study tried to maximize recruitment of the dependent lung and to minimize overdistension of the nondependent lung areas. This approach might have improved the outcomes by minimizing the factors triggering ventilator induced lung injury. It is worth to note that EIT is currently the only method capable of assessing regional overdistension at the bedside.

Two recent prospective studies used EIT to titrate PEEP in ARDS patients [19, 35]. Coincide with the results in these previous studies, we proved that PEEP titration with EIT can significantly improved PaO_2_/FiO_2_, APACHE II score, driving pressure and Crs (Table 2). One limitation of the previous studies was that no control groups were available, so that no clinical outcomes could be compared [19, 35]. Many reasons restricted prospective outcome studies of EIT-guided ventilation. To compare clinical outcome such as weaning success rate, mortality rate, a large number of subjects are required. However, the number of severe ARDS patients is limited due to the improvements in prevention of ventilator-associated lung injury (e.g., lung protective ventilation) and diseases treatment. The control group in the present study was retrospectively analyzed. Lack of randomization was the most relevant limitation in the present study. This may decrease the statistical power of the present findings by introducing bias in both groups. Besides, PEEP selection is only one of the potential factors that are accountable for survival rate. Bias in baseline parameters might have influence on the findings. Ventilator-free days as an endpoint can be misleading [36]. Similarly, the length of ICU and hospital stay can also be ambiguous. Therefore, these parameters were not presented as an outcome. It is noted that patients were predominantly treated in supine position (Table 3). It is known that prone position may lead to increase in oxygenation and decrease in driving pressure [37], and it was commonly used in our hospital in early years. However, as stated in [37], prone positioning requires much more manpower and care. With the rapidly increasing numbers of treated patients, it became an issue in our department and prone positioning could not always be provided. We also acknowledge that the plateau pressure was slightly higher than the recommended 30 cmH_2_O in the Surviving Sepsis Campaign Guideline 2012 [38]. Since a tidal volume of 6 ml/kg predicted body weight was aimed and the initial PEEP was not very high, a high baseline plateau pressure only indicated that the initial compliance of the patients’ lungs was very low. Although patients included in our study were in septic shock, the levels of plateau pressure were high in both groups, and only one was treated in prone position, the mortality rate in our cohort was not higher than the average rate reported [39]. In fact, the mortality rate in the EIT group was lower than the average reported.

The survival rate was not statistically different due to the limited number of patients in both groups. According to the Chi-square distribution, doubling the sample size would result in significant difference if the survival rates stayed the same. Since no previous studies have examined the outcomes comparing EIT-guided PEEP titration and other traditional methods, the effect sizes of the outcome parameters were unknown. No a priori power analysis could be performed. Findings of the present pilot study provided information regarding the deviation of parameter values, which can be used to calculate the sample sizes in future multi-center randomized studies. To reach the statistical power of 80% and a type-I error of 0.05, the sample size should be 113 in each group given the survival rates found in this pilot study. The difference in survival rate is surprisingly big between the EIT and control groups (66.7% vs. 48.4%). The following potential reasons were not examined, which is a major limitation of the present study. (1) Age and the use of nitric oxide inhalation were significantly different in the EIT and control groups. Baseline plateau pressure was in average 1.5 cmH_2_O higher in the EIT group. This finding would probably rather have had a negative influence on outcomes, if any. (2) Although the same protective ventilation strategies and standard care were provided to both groups, potential unknown treatment differences may have influenced the outcomes. Besides, the longer the ICU stay, the more uncertainty exists regarding the factors affecting the survival rates in the two groups. Figure 3 indicated that the highest difference in survival rate occurred at the beginning of ICU stay, when the standard care was comparable in both groups. (3) The incremental/decremental PEEP trial and the low-flow maneuver as two different types of recruitment maneuvers, might have different effectiveness in lung recruitment in the EIT and control groups, which was not examined. (4) As indicated in the Additional file 1, causes of ARDS were different in study subjects with possible unknown effects on mortality.

## Conclusion

In severe ARDS patients, it was feasible and safe to guide PEEP titration with EIT at the bedside. As compared with pressure–volume curve, the EIT-guided PEEP titration may be associated with improved oxygenation, compliance, driving pressure, and weaning success rate. The findings encourage further randomized control study with a larger sample size and potentially less bias in the baseline data.

## Additional file

**Additional file 1.** Detailed demographics and individual diagnoses of patients in both EIT and control groups.

## Abbreviations

APACHE: acute physiology and chronic health evaluation
ARDS: acute respiratory distress syndrome
Crs: respiratory system compliance
ECMO: extracorporeal membrane oxygenation
EIT: electrical impedance tomography
FiO_2_: fractional inspired oxygen
ICU: intensive care unit
LIP: lower inflection point
NMBA: neuromuscular blocking agent
PaO_2_: arterial oxygen partial pressure
PEEP: positive end-expiratory pressure
