# Supplementary material for: Positive end-expiratory pressure titration with electrical impedance tomography and pressure–volume curve in severe acute respiratory distress syndrome
Source: Ann Intensive Care. 2019 Jan 17;9:7. doi: 10.1186/s13613-019-0484-0 (PMC6336593; doi:10.1186/s13613-019-0484-0)
Supplement: Supplementary file 1 — Additional file 1. Detailed demographics and individual diagnoses of patients in both EIT and control groups. [file 13613_2019_484_MOESM1_ESM.docx]

**Online supplement**

**Positive end-expiratory pressure titration with electrical impedance tomography and pressure-volume curve in severe acute respiratory distress syndrome**

Zhanqi Zhao, Mei-Ying Chang, Mei-Yun Chang, Chien-Hung Gow, Jia-Hao Zhang, Yeong-Long Hsu, Inez Frerichs, Hou-Tai Chang, and Knut Möller

Table 1. Demographics of patients in the EIT group

| Pat. No. | Age (yr) | Gender | BP  (mmHg) | bun  (mg/dl) | cr  (mg/dl) |  | Diagnosis | PaO_2_/FiO_2_  (mmHg) | APACHE II |
| --- | --- | --- | --- | --- | --- | --- | --- | --- | --- |
| 1 | 53 | M | 130/69 | 21 | 0.62 |  | Pneumonia, shock, H3N2 influenza A infection | 62 | 22 |
| 2 | 30 | F | 110/78 | 8 | 154 |  | Acute pancreatitis, acute renal failure, acute lung edema | 74 | 17 |
| 3 | 77 | F | 90/56 | 12 | 0.24 |  | Diabetes mellitus without complications, type II | 62 | 34 |
| 4 | 60 | F | 104/70 | 30 | 0.26 |  | Pneumonia | 99 | 15 |
| 5 | 38 | F | 125/80 | 31 | 2.52 |  | Pancreatitis | 95 | 18 |
| 6 | 59 | M | 102/78 | 5 | 0.73 |  | Pneumonia | 86 | 20 |
| 7 | 53 | M | 90/60 | 47 | 2.35 |  | Obstructive chronic bronchitis with acute exacerbation | 62 | 33 |
| 8 | 39 | F | 144/106 | 16 | 0.36 |  | Aneurysm rupture | 38 | 24 |
| 9 | 51 | M | 98/52 | 15 | 0.72 |  | Chronic lymphocytic thyroiditis | 70 | 20 |
| 10 | 55 | F | 114/70 | 27 | 0.60 |  | Acute respiratory failure (H1N1) | 64 | 20 |
| 11 | 52 | M | 112/80 | 82 | 2.57 |  | Acute respiratory failure (H1N1) | 62 | 20 |
| 12 | 28 | M | 96/56 | 16 | 0.52 |  | Acute respiratory failure (H1N1) | 61 | 25 |
| 13 | 39 | M | 102/65 | 15 | 0.40 |  | Pneumonia | 64 | 22 |
| 14 | 36 | F | 145/76 | 10 | 0.60 |  | Pneumonia | 82 | 16 |
| 15 | 59 | M | 144/66 | 36 | 1.94 |  | Pneumonia | 84 | 27 |
| 16 | 52 | M | 107/32 | 44 | 1.99 |  | Pneumonia | 62 | 15 |
| 17 | 60 | M | 103/70 | 42 | 1.83 |  | Pneumonia | 61 | 29 |
| 18 | 72 | M | 147/54 | 55 | 2.43 |  | Pneumonia | 56 | 35 |
| 19 | 54 | M | 145/77 | 16 | 0.56 |  | ICH, IVH | 93 | 20 |
| 20 | 34 | M | 147/54 | 23 | 1.77 |  | Pneumonia | 97 | 20 |
| 21 | 31 | F | 128/74 | 34 | 0.90 |  | OHCA, suspected amniotic fluid embolism | 83 | 37 |
| 22 | 55 | M | 125/80 | 27 | 0.58 |  | Pneumonia | 93 | 21 |
| 23 | 63 | F | 110/70 | 29 | 0.53 |  | Pneumonia, R/O influenza | 46 | 19 |
| 24 | 62 | M | 125/72 | 84 | 1.06 |  | Pneumonia | 64 | 27 |
| Mean | 50.5 | M/F | 119/69 | 27 | 0.73 |  |  | 71.7 | 23.2 |
| SD | 13.3 | 15/9 | 19/14 | 23 | 1.42 |  |  | 16.6 | 6.4 |

Pat. No.: patient number; M: male; F: female; BP: blood pressure; RF: renal function; bun: blood urea nitrogen; cr: creatinine; ICH: intracranial hemorrhage; IVH: intra-ventricle hemorrhage; OHCA: out-of-hospital cardiac arrest; APACHE: acute physiology and chronic health evaluation; PaO_2_/FiO_2_: ratio of arterial partial pressure of oxygen and fraction of inspired oxygen; For renal function, summary data are presented in median and interquartile range. Septic shock was present in every patient.

Table 2. Demographics of patients in the control group

| Pat. No. | Age (yr) | Gender | BP  (mmHg) | bun  (mg/dl) | cr  (mg/dl) | Diagnosis | PaO_2_/FiO_2_  (mmHg) | APACHE II |
| --- | --- | --- | --- | --- | --- | --- | --- | --- |
| 1 | 40 | M | 86/53 | 98 | 9.44 | Pneumonia | 68 | 28 |
| 2 | 44 | M | 124/80 | 36 | 3.52 | Acute aortic dissection s/p, acute kidney injury, shock | 45 | 23 |
| 3 | 19 | F | 111/60 | 9 | 0.80 | Trauma, OHCA | 80 | 33 |
| 4 | 72 | M | 96/44 | 23 | 2.23 | Pneumonia, pulmonary edema | 89 | 30 |
| 5 | 75 | M | 110/51 | 128 | 9.69 | HCC s/p L.C., ARF | 90 | 35 |
| 6 | 72 | M | 84/59 | 33 | 0.80 | Pneumonia | 97 | 24 |
| 7 | 71 | M | 89/41 | 41 | 0.73 | Acute lung edema, AKI | 71 | 21 |
| 8 | 84 | M | 81/42 | 28 | 1.23 | Pneumonia with ARF, CAD-3VD | 42 | 34 |
| 9 | 75 | M | 154/92 | 68 | 4.02 | Pneumonia, AKI, CAD | 53 | 22 |
| 10 | 80 | F | 206/104 | 13 | 0.48 | ICH | 70 | 18 |
| 11 | 48 | M | 118/78 | 47 | 1.13 | STEMI | 94 | 15 |
| 12 | 62 | M | 117/68 | 32 | 1.57 | MODS, AKI | 70 | 17 |
| 13 | 54 | M | 70/36 | 22 | 1.89 | COPD, sepsis, pneumonia | 71 | 14 |
| 14 | 78 | M | 63/33 | 41 | 2.96 | Pneumonia | 75 | 26 |
| 15 | 79 | F | 151/ 63 | 81 | 2.27 | Pneumonia, gastrointestinal bleeding | 52 | 27 |
| 16 | 79 | F | 110/68 | 46 | 2.47 | Pneumonia, AKI | 97 | 27 |
| 17 | 40 | M | 90/68 | 18 | 0.98 | Septic shock, traumatic liver laceration | 77 | 18 |
| 18 | 78 | M | 97/65 | 82 | 3.51 | Gastrointestinal bleeding | 81 | 20 |
| 19 | 83 | F | 137/79 | 123 | 3.47 | Pneumonia, bronchiectasis, Lower gastrointestinal bleeding | 51 | 43 |
| 20 | 71 | M | 106/79 | 76 | 2.72 | Pneumonia | 54 | 26 |
| 21 | 57 | M | 131/71 | 33 | 4.37 | Pneumonia | 98 | 21 |
| 22 | 52 | M | 122/77 | 33 | 1.47 | IHCA, pneumonia, tongue cancer | 66 | 28 |
| 23 | 54 | F | 101/74 | 31 | 0.96 | CAD 2VD, STEMI | 68 | 10 |
| 24 | 64 | F | 119/91 | 110 | 6.99 | Pneumonia | 67 | 28 |
| 25 | 80 | F | 89/59 | 47 | 1.45 | Pneumonia | 60 | 35 |
| 26 | 60 | M | 144/68 | 25 | 1.04 | Pneumonia, short bowel syndrome, SMAT | 75 | 24 |
| 27 | 41 | M | 93/67 | 45 | 1.47 | Heart failure | 53 | 8 |
| 28 | 25 | M | 84/45 | 18 | 1.27 | Traumatic left lung injury | 56 | 30 |
| 29 | 66 | M | 120/64 | 17 | 0.63 | Pneumonia | 64 | 21 |
| 30 | 84 | M | 119/68 | 22 | 0.50 | IPF, pneumonia, COPD | 42 | 24 |
| 31 | 20 | F | 125/79 | 9 | 0.44 | Liver laceration s/p, TRALI | 68 | 24 |
| Mean | 61.5 | M/F | 111/65 | 33 | 1.47 |  | 69.7 | 23.5 |
| SD | 19.2 | 22/9 | 29/17 | 46 | 2.51 |  | 15.9 | 6.9 |

Pat. No.: patient number; M: male; F: female; BP: blood pressure; RF: renal function; bun: blood urea nitrogen; cr: creatinine; OHCA: out-of-hospital cardiac arrest; HCC s/p L.C.: hepatocellular carcinoma status post liver cirrhosis; ARF: acute renal failure; AKI: acute kidney injury; CAD: coronary artery disease; VD: vessel disease; ICH: intracranial hemorrhage; IHCA: in-hospital cardiac arrest; STEMI: ST-segment elevation myocardial infarction. MODS: multiple organ dysfunction syndrome; COPD: chronic obstructive pulmonary disease; SMAT: superior mesenteric arterial thrombosis; IPF: idiopathic pulmonary fibrosis; TRALI: transfusion-related acute lung injury; APACHE II: acute physiology and chronic health evaluation; PaO_2_/FiO_2_: ratio of arterial partial pressure of oxygen and fraction of inspired oxygen; For renal function, summary data are presented in median and interquartile range. Septic shock was present in every patient.
